# Thermogenesis-independent metabolic benefits conferred by isocaloric intermittent fasting in *ob*/*ob* mice

**DOI:** 10.1038/s41598-019-39380-2

**Published:** 2019-02-21

**Authors:** Yun Hye Kim, Ju Hee Lee, Joanna Lan-Hing Yeung, Eashita Das, Ri Youn Kim, Yanqing Jiang, Joon Ho Moon, Hyerin Jeong, Nikita Thakkar, Joe Eun Son, Natasha Trzaskalski, Chi-chung Hui, Kyung-Oh Doh, Erin E. Mulvihill, Jae-Ryong Kim, Kyoung-Han Kim, Hoon-Ki Sung

**Affiliations:** 1 Translational Medicine Program, The Hospital for Sick Children, Toronto, Ontario Canada; 2 Department of Laboratory Medicine and Pathobiology, University of Toronto, Toronto, Ontario Canada; 3 Department of Microbiology, Siliguri College, North Bengal University, West Bengal, India; 4 University of Ottawa Heart Institute, Ottawa, Ontario Canada; 5 Department of Cellular and Molecular Medicine, University of Ottawa, Ottawa, Ontario Canada; 6 Developmental and Stem Cell Biology Program, The Hospital for Sick Children, Toronto, Ontario Canada; 7 Department of Biochemistry, Microbiology and Immunology, University of Ottawa, Ottawa, Ontario Canada; 8 Department of Molecular Genetics, University of Toronto, Toronto, Ontario Canada; 9 Department of Physiology, College of Medicine, Yeungnam University, Gyeongsan, Republic of Korea; 10 Department of Biochemistry and Molecular Biology & Smart-aging Convergence Research Center, College of Medicine, Yeungnam University, Gyeongsan, Republic of Korea; 11 Banting and Best Diabetes Centre, University of Toronto, Toronto, Ontario Canada

## Abstract

Intermittent fasting (IF) is an effective dietary intervention to counteract obesity-associated metabolic abnormalities. Previously, we and others have highlighted white adipose tissue (WAT) browning as the main underlying mechanism of IF-mediated metabolic benefits. However, whether IF retains its efficacy in different models, such as genetically obese/diabetic animals, is unknown. Here, leptin-deficient *ob/ob* mice were subjected to 16 weeks of isocaloric IF, and comprehensive metabolic phenotyping was conducted to assess the metabolic effects of IF. Unlike our previous study, isocaloric IF-subjected *ob/ob* animals failed to exhibit reduced body weight gain, lower fat mass, or decreased liver lipid accumulation. Moreover, isocaloric IF did not result in increased thermogenesis nor induce WAT browning in *ob/ob* mice. These findings indicate that isocaloric IF may not be an effective approach for regulating body weight in *ob/ob* animals, posing the possible limitations of IF to treat obesity. However, despite the lack of improvement in insulin sensitivity, isocaloric IF-subjected *ob/ob* animals displayed improved glucose tolerance as well as higher postprandial insulin level, with elevated incretin expression, suggesting that isocaloric IF is effective in improving nutrient-stimulated insulin secretion. Together, this study uncovers the insulinotropic effect of isocaloric IF, independent of adipose thermogenesis, which is potentially complementary for the treatment of type 2 diabetes.

## Introduction

Over the past few decades, the prevalence of obesity has dramatically increased across all genders and age groups, reaching a global epidemic level. As obesity is strongly associated with the development of other chronic health conditions, such as type 2 diabetes, hypertension, and non-alcoholic fatty liver disease (NAFLD), development of feasible and practical treatments to counteract obesity is urgently needed. A number of factors contribute to obesity, including genetic determinants, environmental and behavioural traits^1–3^. In particular, polymorphisms in various genes regulating appetite and metabolic rate were identified to predispose individuals to obesity.

Leptin (encoded by *ob* gene) is an adipokine that plays a critical role in energy homeostasis, appetite, and weight regulation^4–6^. The mouse model of leptin deficiency with *ob* gene mutation (*i.e. ob/ob* mice) displays severe metabolic abnormalities, such as hyperphagia, hyperglycemia, and obesity at an early age, serving as a genetic model for obesity^7^. Although leptin gene mutations are rare in human obesity^8^, the severe and early-onset metabolic dysfunctions seen in *ob*/*ob* mice present an ideal model to study the efficacy of various therapeutic approaches to combat obesity and associated metabolic disorders.

Fasting, characterized by periods of food deprivation for several hours to a few days, is a popular dietary approach for weight management in humans^9^. In addition, the beneficial effects of fasting on ageing, cancer, cardiovascular diseases, and neurodegenerative diseases have been well documented in both animals and humans^10–13^. As such, various dietary interventions adapting fasting regimens, such as intermittent fasting (IF) and the fasting-mimicking diet, have gained popularity as therapeutic modalities against obesity. Importantly, multiple studies have demonstrated that limiting the caloric intake duration to a shorter time-window without changing the diet quantity or quality can bring significant metabolic benefits^14–16^. This suggests that even in the absence of caloric reduction, modification of the eating pattern can sufficiently improve metabolic health. Thus, isocaloric IF can serve as a simpler nutritional regulation method, compared to prolonged fasting and caloric reduction^17,18^. Recently, we and others have demonstrated that the metabolic effects of IF in mice are mediated by multiple underlying mechanisms^17,19^. These studies showed that IF improves metabolic homeostasis by ameliorating diet-induced obesity and associated metabolic dysfunctions, with a reduction of body weight gain, improvement in glucose tolerance and insulin sensitivity, and hepatic lipid clearance. These metabolic benefits were primarily achieved by beige fat formation in the white adipose tissue (WAT), which is driven via vascular endothelial growth factor (VEGF)-dependent anti-inflammatory macrophage activation^17^ and/or via selective elevation of acetate/lactate metabolites from the gut microbiota^19^. However, whether IF-mediated metabolic benefits including reductions in obesity and improvements in glucose metabolism are entirely attributed to WAT browning is not well understood. Alternatively, proliferation of neurogenin-3 positive (Ngn3+) pancreatic β cells during fasting-mimicking diet^20^ or systemic changes in autophagy by time-restricted feeding may contribute to the whole-body benefits gained by various fasting regimens^21^, in addition to WAT browning. Moreover, although previous studies have shown beneficial effects of IF in diet-induced obese mice, it is still unclear whether IF retains its benefits in genetically obese/diabetic models, especially under isocaloric conditions. Therefore, to test this, we performed isocaloric IF regimen in *ob*/*ob* mice, as recently reported^17^. Investigating whether IF-mediated benefits are sustained or compromised in these subjects may allow us to better strategize fasting regimens for obese/diabetic patients with a genetic predisposition.

## Results

### Isocaloric intermittent fasting fails to reduce body weight gain and fat mass in *ob/ob* mice

**Figure 1:**
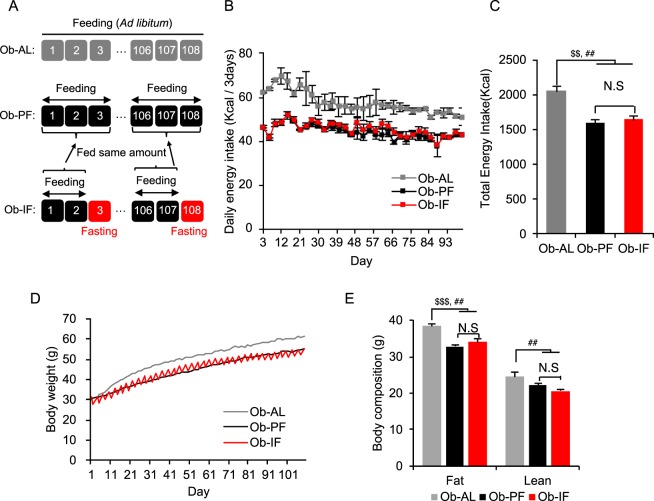
Isocaloric intermittent fasting does not modify body weight in *ob/ob* mice. (**A**) An experiment scheme of the 2:1 intermittent fasting (IF) regimen. (**B**) Daily energy intake during 16 weeks of IF cycles. (**C**) Total energy intake during 16 weeks of IF cycles. (**D**) Body weight measurement of *ob/ob* mice for 16 weeks. (**E**) Body composition of fat and lean mass in *ob/ob* mice subjected to the *ad libitum* (Ob-AL), *ob/ob* mice subjected to the pair-fed *ad libitum* (Ob-PF) and *ob/ob* mice subjected to the intermittent fasting (Ob-IF). Data are expressed as mean ± s.e.m. (Ob-AL: n = 4; Ob-PF: n = 7; Ob-IF: n = 6); Ob-AL vs. Ob-PF: ^$^P < 0.05; Ob-AL vs. Ob-IF: ^#^P < 0.05; Ob-PF vs. Ob-IF: *P < 0.05.

6-week old male *ob*/*ob* mice were subjected to 16 weeks of 2:1 IF (2 days of feeding – 1 day of fasting) (Fig. 1A). We have previously reported that 2:1 IF regimen provides fasted wild-type mice sufficient time to compensate for the food intake deficit after 1 day of fasting, such that the food intake is equal to the level of *ad libitum* (AL) animals^17^. However, unlike our previous study in wild-type mice, *ob/ob* mice subjected to 2:1 IF (Ob-IF; 1635 Kcal/16 weeks) exhibited mild (21%) reductions in total food intake, compared to the AL group (Ob-AL; 2066 Kcal/16 weeks) (Fig. 1B,C), due to hyperphagic behaviours of *ob/ob* mice. Hence, to solely test the metabolic effects of IF in the absence of caloric reduction, a pair-fed group (Ob-PF) that maintains the same caloric intake as the Ob-IF was employed as an additional control group. Despite mildly lower food intake than Ob-AL mice, Ob-PF mice still exhibited hyperphagic obesity with approximately 20% higher energy intake and 25% higher body weight (~50 g) as well as metabolic dysfunction, compared to our previous high-fat diet-induced obese mouse model (~40 g) (Supplementary Table 1)^17^. This suggests that Ob-PF mice serve as an adequate obese and metabolically unhealthy model to test the metabolic benefits of IF under isocaloric condition. Therefore, although Ob-AL mice serve as a standard obese/diabetic model^22^, our current study mainly focuses on the comparison between Ob-PF and Ob-IF group. In contrast to our previous study using the pair-fed wild-type control mice^17^, 16 weeks of isocaloric IF regimen in *ob/ob* mice did not reduce body weight gain, compared to Ob-PF, while both Ob-IF and Ob-PF mice exhibited a significantly lower body weight than Ob-AL mice (Fig. 1D). Ob-IF and Ob-PF groups also exhibited comparable fat and lean mass but were significantly reduced compared to Ob-AL mice (Fig. 1E). These findings suggest that isocaloric IF failed to induce caloric-independent decrease in body weight in *ob/ob* mice, and that reduced body weight in Ob-IF group compared to Ob-AL are largely due to a mild decrease in caloric intake, not by repeated fasting-feeding cycles as seen in wild-type mice.

### Isocaloric intermittent fasting does not reduce adipocyte size nor ameliorate hepatic lipid accumulation in *ob*/*ob* mice

**Figure 2:**
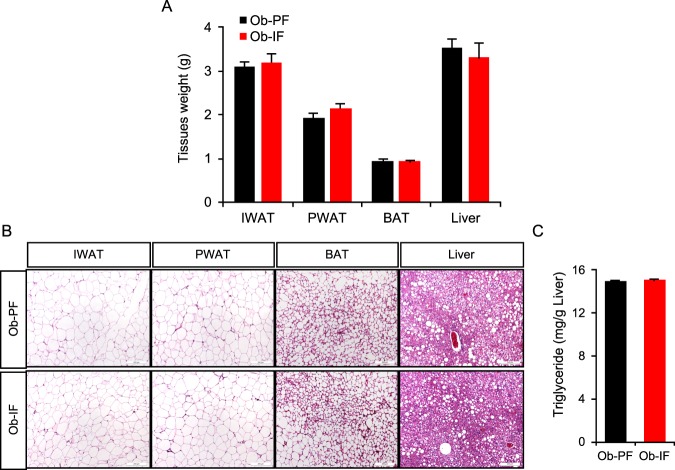
Isocaloric intermittent fasting does not reduce fat mass nor improve fatty liver phenotype in *ob/ob* mice. (**A**) Tissue weights of inguinal and perigonadal white adipose tissue (IWAT and PWAT, respectively), brown adipose tissue (BAT), and liver. (**B**) H&E stained sections of IWAT, PWAT, BAT and liver. (**C**) Liver triglyceride level. Data are expressed as mean ± s.e.m. (Ob-PF: n = 6–7; Ob-IF: n = 5–6).

Our previous study has highlighted the selective reduction in sizes of adipose depots and adipocytes without affecting lean mass in IF-subjected mice^17^. However, body composition analysis revealed no significant difference in both total fat and lean mass between the Ob-IF and Ob-PF groups after 16 weeks of IF (Fig. 1E). Consistently, Ob-IF mice did not exhibit a reduction in perigonadal and inguinal WAT (PWAT and IWAT, respectively) weights (Fig. 2A), and there was no observable difference in adipocyte size in WAT depots and lipid accumulation in brown adipose tissue (BAT), compared to Ob-PF mice (Fig. 2B). Importantly, we did not detect any beige adipocytes in WAT from either group. Lastly, IF was not able to prevent liver steatosis, indicated by comparable liver weight (Fig. 2A) as well as lipid accumulation assessed by histology and triglyceride (TG) content measurement (Fig. 2B,C). These findings suggest that IF is not effective in rescuing obese phenotypes in *ob*/*ob* mice under isocaloric condition, in contrast to what was observed in pair-fed study in wild-type mice^17^.

### Isocaloric intermittent fasting improves glucose homeostasis in *ob*/*ob* mice

**Figure 3:**
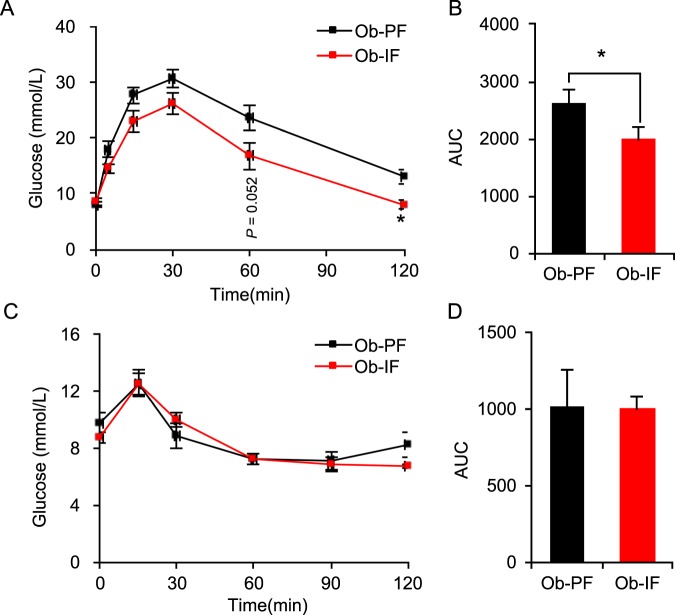
Isocaloric intermittent fasting partially improves glucose homeostasis in *ob/ob* mice. (**A**) Glucose tolerance test (GTT) in Ob-PF (n = 12) and Ob-IF (n = 11) mice. (**B**) Quantification of the area under the curve (AUC) from the IPGTT. (**C**) Insulin tolerance test (ITT). (**D**) Quantification of AUC from the IPITT. Data are expressed as mean ± s.e.m. (Ob-PF: n = 6–7; Ob-IF: n = 5–6); Ob-PF vs. Ob-IF: *P < 0.05.

One of the significant metabolic advantages achieved by isocaloric IF is an improvement in glucose homeostasis. Specifically, IF markedly enhances glucose handling capacity and insulin sensitivity in wild-type mice^17^. Notably, despite the failures in IF-mediated weight gain reduction, we found that Ob-IF animals displayed significant improvement in glucose handling, as shown with smaller glucose excursions in glucose tolerance test (GTT), compared to Ob-PF mice (Fig. 3A,B). On the other hand, insulin sensitivity, indicated by the insulin tolerance test (ITT) was not improved by IF in *ob*/*ob* mice, compared to Ob-PF mice (Fig. 3C,D). These data suggest that isocaloric IF can improve glucose homeostasis in *ob/ob* mice, which does not originate from the differences in body weight and insulin sensitivity.

### Isocaloric intermittent fasting does not stimulate adipose thermogenesis in *ob*/*ob* mice

**Figure 4:**
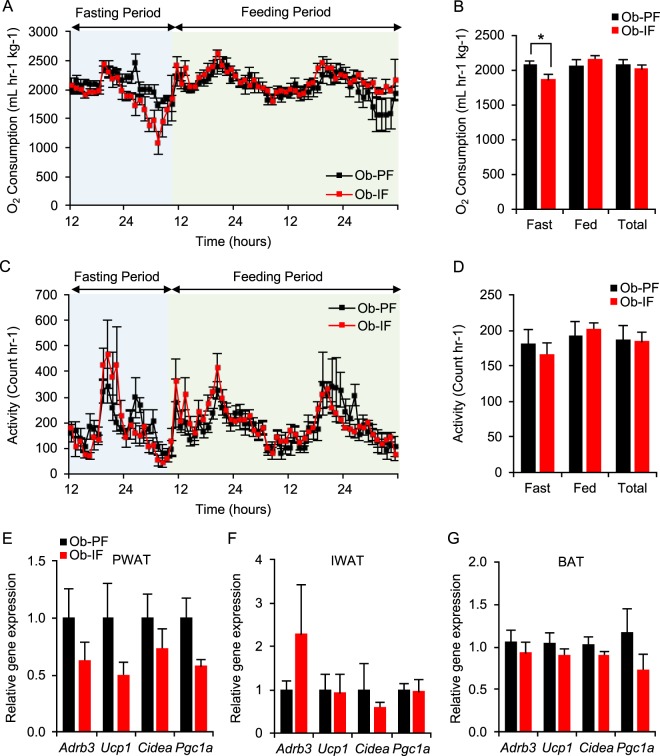
Isocaloric intermittent fasting does not stimulate adipose thermogenesis  in *ob/ob* mice. (**A**) The change of O_2_ consumption normalized by body weight and (**B**) average of O_2_ consumption per hour during fasting and feeding periods, and combined. (**C**) The change of activity and (**D**) average of physical activities per hour during fasting and feeding periods, and combined. No changes in browning marker gene expression in (**E**) PWAT, (**F**) IWAT, and (**G**) BAT of Ob-IF. Data are expressed as mean ± s.e.m. (Ob-PF: n = 7; Ob-IF: n = 6); Ob-PF vs. Ob-IF: *P < 0.05.

Our recent study and others have demonstrated that IF-mediated metabolic benefits are primarily attributed to an increase in energy expenditure via adipose thermogenesis, particularly browning of the WAT^17,19^. In addition, while *ob/ob* mice have been thought to be thermogenically limited, a previous study using caloric restriction suggests that *ob/ob* mice can induce adipose thermogenesis^22^. We therefore examined the thermogenic effect of isocaloric IF in *ob/ob* mice as a potential mechanism of improved glucose homeostasis. However, our indirect calorimetry analysis revealed no significant difference in energy expenditure, measured by the oxygen consumption rate (VO_2_), between Ob-PF and Ob-IF animals, despite a reduction in VO_2_ during fasting periods in Ob-IF animals (Fig. 4A,B). In addition, no significant changes in activity were observed in Ob-IF mice, compared to Ob-PF mice (Fig. 4C,D). Consistent with these findings, the expression of *Adrb3* (*i.e*. indicative of activated sympathetic tone) and beige/brown adipose markers (*i.e. Ucp1*, *Cidea, Ppargc1a*) were comparable between Ob-IF and Ob-PF animals in all adipose depots, including PWAT, IWAT and BAT (Fig. 4E–G). Together, these data suggest that IF failed to induce adipose thermogenesis in *ob*/*ob* mice under isocaloric condition.

### Isocaloric intermittent fasting does not modify adipose-derived factors and inflammatory gene expression in *ob/ob* mice

**Figure 5:**
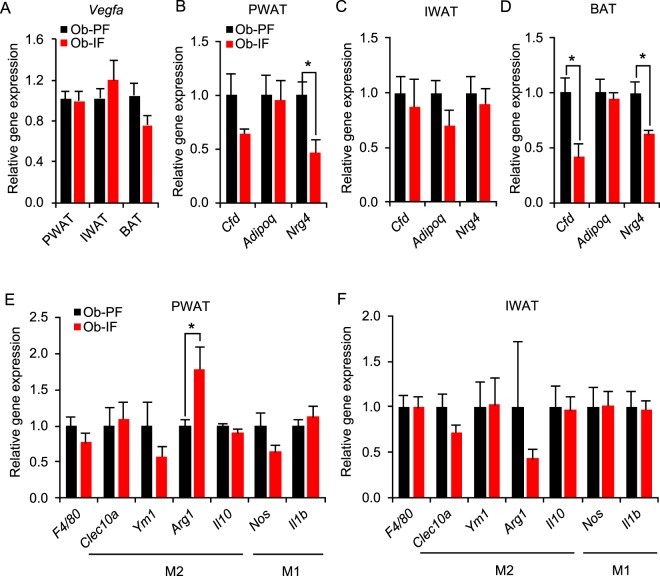
Isocaloric intermittent fasting does not modify expression levels of adipose-derived factor and inflammatory genes in *ob/ob* mice. (**A**) No changes in *Vegfa* mRNA expression in PWAT, IWAT, and BAT. mRNA expression levels of *Cfd*, *Adipoq*, and *Nrg4* in (**B**) PWAT, (**C**) IWAT, and (**D**) BAT. Inflammatory marker gene expression analysis in (**E**) PWAT and (**F**) IWAT. Data are expressed a mean ± s.e.m. (Ob-PF: n = 7; Ob-IF: n = 6); Ob-PF vs. Ob-IF: *P < 0.05.

Our previous study has demonstrated that IF leads to elevated expression and secretion of various adipose-derived factors which play a protective role against diet-induced obesity and metabolic abrnormalities^17^. Notably, we have illustrated fasting-mediated elevation of adipose-VEGF, accompanied with increased angiogenesis, as the underlying mechanism of IF-induced WAT browning^17^. On the other hand, in the *ob*/*ob* animal model, IF failed to increase *Vegfa* mRNA expression in PWAT, IWAT, and BAT, consistent with the lack of IF-induced adipose thermogenesis (Fig. 5A). In addition, the gene expression of other beneficial adipose-derived factors that are upregulated by IF in wild-type mice^17^, such as *Adipoq* (adiponectin), *Cfd* (adipsin), and *Nrg4* (neuregulin 4), were unchanged or even reduced in PWAT, IWAT and BAT of Ob-IF mice, compared to those of Ob-PF mice (Fig. 5B–D). Moreover, the expression levels of various macrophage-associated genes in PWAT and IWAT (*e.g. F4/80* for pan-macrophage, *Clec10* and *Il10* for anti-inflammatory M2 macrophage, *Nos* and *Il1b* for pro-inflammatory M1 macrophage) were unchanged between Ob-IF and Ob-PF mice (Fig. 5E,F). Collectively, our data suggest that isocaloric IF-induced beneficial adipokine and anti-inflammatory change were largely abrogated in *ob*/*ob* mice.

### Isocaloric intermittent fasting enhances insulin and incretin secretions in *ob*/*ob* mice

**Figure 6:**
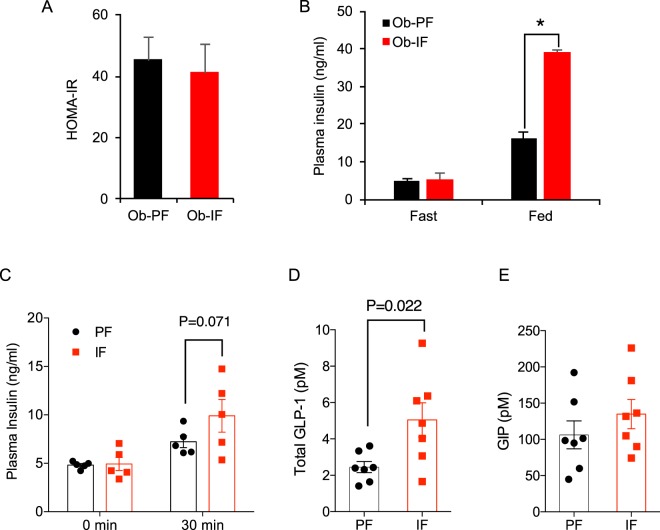
Isocaloric intermittent fasting enhances insulin and incretin production in *ob/ob* mice. (**A**) Homeostatic model assessment of insulin resistance (HOMA-IR). (**B**) The plasma insulin levels of Ob-PF and Ob-IF in fasting and postprandial conditions. (**C**) Glucose-stimulated insulin secretion measured before and 30 min after glucose i.p. injection. (**D**,**E**) The total plasma GLP-1 and GIP levels of Ob-PF and Ob-IF mice in fasting condition. Data are expressed as mean ± s.e.m. (Ob-PF: n = 7; Ob-IF: n = 6); Ob-PF vs. Ob-IF: *P < 0.05.

Unlike our and other previous studies^17,22^, above results suggest that improved glucose homeostasis by isocaloric IF in *ob/ob* mice was not likely mediated by adipose thermogenesis and beneficial adipose-derived factors. Since glucose handling capacity measured by GTT was enhanced by isocaloric IF in *ob/ob* mice (Fig. 3A) without increased insulin sensitivity (Fig. 3C), we postulated that improved GTT is potentially mediated by increased plasma insulin^23^. Therefore, we examined plasma insulin levels in both fasting and postprandial conditions and also computed homeostasis model assessment-estimated insulin resistance (HOMA-IR). Consistent with ITT data (Fig. 3C), HOMA-IR was not improved by IF in *ob*/*ob* mice (Fig. 6A). Notably, while the fasting insulin levels were indistinguishable between Ob-PF and Ob-IF animals, Ob-IF mice displayed significantly increased insulin secretion in response to food intake, as indicated by a fold higher postprandial plasma insulin level, compared to that of Ob-PF mice (Fig. 6B). Similarly, glucose-stimulated insulin secretion was greater in Ob-IF mice as shown in higher plasma insulin levels at 30 minutes post-glucose injection, compared to Ob-PF mice (Fig. 6C). These data suggest that improved glucose homeostasis in IF-subjected *ob/ob* mice was higher insulin secretory response.

Among several insulinotropic factors and pathways, two incretin hormones, glucagon-like peptide-1 (GLP-1) and glucose-dependent insulinotropic polypeptide (GIP) are known to not only potentiate nutrient-stimulated insulin secretion from pancreatic β cells but also preserve pancreatic β cell mass by enhancing β cell proliferation and protecting them from apoptosis^24,25^. Since secretion of GLP-1 and GIP levels are enhanced by food ingestion, we tested whether IF affected incretin levels in *ob*/*ob* mice. Interestingly, fasting plasma total GLP-1 levels were significantly higher in Ob-IF mice, compared to Ob-PF mice (Fig. 6D), while plasma GIP levels were not different between the two groups (Fig. 6E). Together, our data suggest that while isocaloric IF may not be an effective approach for weight control in genetically obese *ob*/*ob* mice, it still confers a partial metabolic benefit with improved glucose handling, potentially through incretin-enhanced insulin production.

## Discussion

The beneficial effects of IF against metabolic diseases have been documented in both humans and animals for decades, yet the underlying mechanisms of IF have only recently been elucidated^17,19^. Specifically, we and others have demonstrated that the metabolic benefits of IF in WT mice are primarily attributed to browning of the WAT, which can increase systemic energy expenditure. However, limited capacity for WAT browning in obese/aged individuals, has been reported in several studies^26,27^, which suggests potential limitations of IF in subjects with unfavourable metabolic conditions. Hence, the aim of this study was to test the efficacy of IF on a model other than conventional diet-induced obese models, by employing genetically obese/diabetic *ob/ob* animals.

In the present study, we found that while isocaloric IF in *ob*/*ob* mice improves glucose handling capacity, other metabolic improvements that were observed in IF-treated wild-type obese mice, such as insulin sensitivity and liver lipid clearance, are largely diminished. Although both IF-treated and pair-fed *ob/ob* mice showed lower body weight gain and increased browning gene expression compared to Ob-AL mice, there was no difference between Ob-IF and Ob-PF mice, which were significantly different in wild-type mice. This discrepancy in metabolic response to IF can be possibly explained as follows: First, leptin is a key adipokine in energy homeostasis and thermogenic activity, whose expression is markedly regulated by fasting and feeding^28–30^. Due to leptin deficiency, *ob*/*ob* mice not only display hyperphagia but also exhibit reduced thermogenic response^31^. In particular, *ob*/*ob* mice develop a severe fasting-induced hypothermia^32^, which along with hyperphagia, can result in increased preservation of body mass and energy expenditure, compared to wild-type animals. In addition, leptin directly impacts on hypothalamic pro-opiomelanocortin (POMC) neurons in promotion of adipose thermogenesis including WAT browning^33^. These observations thus suggest possible mechanisms involved in the impairment of IF-mediated adipose thermogenesis in *ob*/*ob* mice. Secondly, lack of adipose thermogenesis in *ob*/*ob* mice subjected to IF can be related to the insufficient induction of fasting-mediated adipose-VEGF expression that is critical for WAT browning^17^. For example, several previous studies have demonstrated that leptin is an upstream regulator of VEGF expression in endothelial and cancer cells^34–36^. As well, *ob*/*ob* mice display smaller capillary fenestrations and reduced vascular permeability in the adipose tissue^37^. Thus, insufficient fasting-induced adipose-VEGF induction by leptin deficiency may also underlie the lack of WAT browning in IF-treated *ob*/*ob* mice. Third, due to the hyperphagic nature of *ob/ob* mice, 24 hour fasting may be more stressful to them, compared to wild-type mice. Indeed, as stress exaggerates diet-induced obesity through neuropeptide Y (NPY)-mediated pathway in visceral WAT^38^, we observed higher gene expression of NPY receptor (*Npy2r*) in PWAT of Ob-IF mice, compared to Ob-PF mice (data not shown). This suggests a possibility that augmented fasting-mediated stress in the *ob/ob* mice counteracts the potential benefits of IF on body weight loss. Further studies to examine the interaction between NPY and leptin signaling in adipose tissues would be of interest. Lastly, the blunted metabolic effect of IF could be due to abnormal microbiome composition in *ob*/*ob* animals^39^. Gut microbiota exerts a pivotal role in energy metabolism and beige adipogenesis. Previous studies have revealed significantly altered microbiome dynamics during feeding and fasting periods^40,41^. Likewise, microbiota metabolites contribute to induction of beige adipogenesis in WAT by intermittent fasting^19^. These observations suggest that the dysbiotic gut microbiota in *ob*/*ob* mice may also be associated with reduced energy expenditure and perturbed browning capacity of the WAT.

We have shown that IF markedly increases insulin sensitivity and glucose homeostasis in high fat-fed wild-type mice^17^. However, postprandial plasma insulin level in wild-type IF-treated mice was indistinguishable compared to mice *ad libitum*^17^. This suggests that due to the significant elevation in insulin sensitivity by IF, IF-treated wild-type mice do not require augmented insulin production and secretion from the pancreas. On the other hand, improved glucose homeostasis in IF-treated *ob*/*ob* mice in this study was primarily attributed to increased insulin secretion, not insulin sensitivity, suggesting improved β cell function. It is generally accepted that *ob/ob* mice display enlarged pancreatic β cell mass due to increased insulin demand. However, upon chronic hyperglycemia, β cell exhaustion can lead to apoptosis, further exacerbating β cell dysfunction^42^. As recently shown in diabetic mice treated with fasting-mimicking diet^20^, it is possible that increased postprandial insulin secretion in IF-treated *ob*/*ob* mice is mediated by IF-promoted β cell regeneration, preservation or proliferation. While these potential mechanisms^20,43^ await further investigation, our novel finding of IF-induced GLP-1 increase in *ob*/*ob* mice introduces a new potentially important player from the gut in the IF-mediated metabolic benefits. The glucose metabolic phenotype seen in Ob-IF mice is indeed aligned with the action of GLP-1 in glucose metabolism in promoting insulin secretion, not its sensitivity^24,25^. As incretin-based therapeutics, such as GLP-1 receptor (GLP-1R) agonists and dipeptidyl peptidase-4 inhibitors (DPP-4i), are commonly used for treatment of type 2 diabetes, our observation that IF can physiologically increase GLP-1 levels suggests a possibility that IF can be a lifestyle supplement to existing diabetes treatments by increasing the efficacy or reducing the dosages. Further study is warranted to examine the potential mechanisms of IF in regulating glucose homeostasis through incretin secretion.

Collectively, since the blunted effect of IF on insulin sensitivity in *ob*/*ob* mice is likely associated with largely impeded adipose tissue-related metabolic benefits, such as VEGF induction and WAT browning, *ob*/*ob* mouse model is a unique tool to distinguish the effects of IF on glucose homeostasis into insulin sensitivity and production/secretion. Meanwhile, the present study poses the limitation of IF as a therapeutic modality as it may not work equally for all individuals based on their genetic predispositions and metabolic status. For example, low brown fat activity in humans is correlated with age, advanced obesity, and diminished metabolic health^44,45^. Indeed, human IF studies have reported substantial variations in metabolic benefits of IF, depending on the metabolic condition, age and sex of participants^46^. Therefore, further investigations aimed to delineate other mechanisms of IF, particularly for humans who are incapable of adipose tissue browning or are insulin resistant (*e.g*. ageing and diabetes) are warranted. These will provide deeper insights into the efficacy of IF, enabling to develop a defining method of an optimal indication and inclusion criteria in clinical settings.

## Methods

### Animals

All animal experiments were performed in accordance with protocols approved by The Centre for Phenogenomics Animal Care Committee (ACC), and conformed to the standards of the Canadian Council on Animal Care. All mice were housed in standard vented cages in a temperature- and humidity-controlled rooms with 12-hour light-dark cycles (21–22 °C, 30–60% humidity for normal housing), and free access to water. The *ob*/*ob* mice were obtained from the the Jackson Laboratory. In all experiments, only male animals were analyzed.

### Intermittent fasting regimen and diet

As the Ob-IF animals were not able to fully compensate for the food intake to the level of Ob-AL animals, body weight-matched 6-week-old male *ob*/*ob* mice were randomly divided into two groups: pair-fed (PF) and intermittent fasting (IF) groups. Mice were fed with normal chow (Harlan, #2918). Mice in the IF group were subjected to 2:1 IF regimen, comprising 1 day of fasting, followed by 2 days of free feeding. The food was removed at 12:00 PM and provided again the following day (24 h later) at 12:00 PM. Mice in the PF group were fed the same amount of food as the mice in IF group. Specifically, the amount of food as the IF group consumed was splitted into two daily amounts and then provided to the PF group, in order to minimize potential fasting exposure due to the hyperphagic behavior of *ob/ob* mice. No weight loss was observed in Ob-PF mice.

### Body weight and food consumption

Body weight and food consumption were measured before and after fasting periods from 6 to 23 weeks of age. Caloric intake was calculated based on nutritional information (normal chow: 3.3 Kcal/g, 17% fat) provided by the manufacturers. Body composition was analyzed using the body composition analyzer (EchoMRI-100 machine, Echo Medical Systems, Houston, TX, USA), which quantifies fat and lean mass in live, non-anesthetized mice.

### Energy expenditure analysis

Energy metabolism was evaluated through indirect calorimetry (Oxymax System, Columbus Instruments) over a period of 72 h (a single cycle of IF). Oxygen consumption (VO_2_), an indicator of energy expenditure, was normalized by the body weight of individual animals.

### Glucose and insulin tolerance tests

For glucose and insulin tolerance tests, mice were subjected to intraperitoneal injection of glucose (1 mg/g of body weight) or insulin (0.65 mU/g of body weight, Humulin^®^) after fasting 16 hours for GTT and 6 hr for ITT with water *ad libitum*. Blood glucose was measured from the tail at 0, 15, 30, 60, and 120 mins post-glucose, using a glucometer (Contour NEXT, Bayer HealthCare). The homeostatic model assessment of insulin resistance (HOMA-IR) was calculated by using the values of fasting plasma glucose (FPG, mmol/L) and plasma insulin (PI, mU/L) as follows: HOMA-IR = FPG × PI/22.5.

### Histological analysis

For histological analysis, freshly harvested tissues were fixed in 4% paraformaldehyde and embedded in paraffin. Sections of 4–5 µm were stained with haematoxylin and eosin (H&E), and the stained regions were randomly selected for imaging.

### RNA extraction and quantitative PCR analysis

Total RNA was extracted from tissues using RNeasy Lipid Tissue Kit (Qiagen), and complementary DNA was synthesized from 1 µg of RNA using M-MLV reverse transcriptase (Invitrogen) with oligo(dT)_12–18_ primer. Gene expression assay was conducted using SYBR Green methods on Quantstudio 5 (Applied Biosystems), and relative cycle threshold (CT) values were normalized by *36b4* gene. Sequences of qPCR primer sets in this study used are in Supplementary Table 2.

### Plasma analysis

Plasma parameter was measured using samples collected from 16 h-fasted mice or 24 h-postprandial mice. Blood was collected into EDTA-coated tubes and centrifuged at 5000 rpm for 10 minutes at 4 °C to separate plasma. Plasma insulin levels were quantified using an ALPCO Diagnostics enzyme-linked immunosorbent assay (ELISA) kit (ALPCO, 80-INSMS-E01) according to the manufacturer’s instructions. Plasma total GLP-1 levels were measured using a Mesoscale V-Plex Total GLP-1 Assay (Mesoscale, K1503PD-1) and total GIP levels by ELISA (Crystal Chem, 81517) according to manufacturers’ instructions.

### Hepatic lipid analysis

Total triglyceride (TG) were extracted from liver tissues using Folch solution (chloroform-methanol, 2:1 v/v), dried and dissolved in 100% EtOH^47^. Hepatic lipid extracts were assayed for TG levels using commercial assay kits (Randox, TR213).

### Statistical analysis

All results are presented as mean ± s.e.m. Statistical significance of differences among groups was determined by two-tailed unpaired and paired Student’s *t*-test.

## Supplementary information

Supplementary Information
