# Supplementary material for: Thermogenesis-independent metabolic benefits conferred by isocaloric intermittent fasting in ob/ob mice
Source: Sci Rep. 2019 Feb 21;9:2479. doi: 10.1038/s41598-019-39380-2 (PMC6385507; doi:10.1038/s41598-019-39380-2)
Supplement: Supplementary file 1 — Supplementary Information [file 41598_2019_39380_MOESM1_ESM.pdf]

Supplementary Information

Thermogenesis-independent metabolic benefits conferred by isocaloric intermittent fasting in *ob/ob* mice

Yun Hye Kim[1+], Ju Hee Lee[1,2+], Joanna Lan-Hing Yeung[1], Eashita Das[1,3], Ri Youn Kim[4,5], Yanqing Jiang[1], Joon Ho Moon[1], Hyerin Jeong[1], Nikita Thakkar[1], Joe Eun Son[6], Natasha Trzaskalski[4,7], Chi-chung Hui[6,8], Kyung-Oh Doh[9], Erin E. Mulvihill[4,7], Jae-Ryong Kim[10*], Kyoung-Han Kim[4,5*], Hoon-Ki Sung[1,2,11*]

[1]Translational Medicine Program, The Hospital for Sick Children, Toronto, Ontario, Canada; [2]Department of Laboratory Medicine and Pathobiology, University of Toronto, Toronto, Ontario, Canada; [3]Department of Microbiology, Siliguri College, North Bengal University, West Bengal, India; [4]University of Ottawa Heart Institute, Ottawa, Ontario, Canada; [5]Department of Cellular and Molecular Medicine, University of Ottawa, Ottawa, Ontario, Canada; [6]Developmental and Stem Cell Biology Program, The Hospital for Sick Children, Toronto, Ontario, Canada; [7]Department of Biochemistry, Microbiology and Immunology, University of Ottawa, Ottawa, Ontario, Canada; [8]Department of Molecular Genetics, University of Toronto, Toronto, Ontario, Canada; [9]Department of Physiology and [10]Department of Biochemistry and Molecular Biology & Smart-aging Convergence Research Center, College of Medicine, Yeungnam University, Republic of Korea; [11]Banting and Best Diabetes Centre, University of Toronto, Toronto, Ontario, Canada.

Correspondence:
Jae-Ryong Kim, Tel: 82-53-620-4342, E-mail: kimjr000@gmail.com
Kyoung-Han Kim, Tel: 613-696-7222, E-mail: hankim@uottawa.ca
Hoon-Ki Sung, Tel: 416-813-7654 ext.309430, E-mail: hoon-ki.sung@sickkids.ca

Supplementary Table 1. Comparison of ND-AL, HFD-AL and Ob-PF

Metabolic profile	ND-AL	HFD-AL	Ob-PF
Body Weight (g)	32.5 ± 0.5	43.2 ± 2.1$^{\$\$\$}$	55.5 ± 0.9$^{\#\#\#***}$
Total fat weight (g)	6.8 ± 0.9	16.7 ± 2.1$^{\$\$\$}$	32.9 ± 0.4$^{\#\#\#***}$
Lean weight	25.3 ± 0.8	25.7 ± 0.8	22.2 ± 0.6$^{\#**}$
Fasting glucose (mmol/L)	5.1 ± 0.3	5.6 ± 0.6	8.0 ± 0.5$^{\#\#*}$
HOMA-IR	N/A	12.8 ± 5.9	45.4 ± 7.1**
IPGTT AUC	1215.6 ± 44.3	2064.6 ± 190.3$^{\$}$	2638.6 ± 180.7$^{\#\#\#\#*}$
IPITT AUC	902.5 ± 72.3	896.8 ± 85.2	1019.6 ± 99.8

ND-AL: normal diet fed *ad libitum* (n=7); HFD-AL: high fat diet (45%) fed *al libitum* (n=6); Ob-PF: pair-fed *ob/ob* mice (n=7). Statistical analysis was performed by ANOVA test.
$: P<0.05, $$: P<0.01, $$$: P<0.005 ND-AL vs HFD-AL,
#: P<0.05, ##: P<0.01, ###: P<0.005 ND-AL vs Ob-PF,
*: P<0.05, **: P<0.01, ***: P<0.005 HFD-AL vs Ob-PF.

Supplementary Table 2. List of real-time PCR primer sequences

**Gene**	**Forward**	**Reverse**
Vegfa	TGAAGCCCTGGAGTGCGT	AGGTTTGATCCGCATGATCTG
Cfd	CATGCTCGGCCCTACATGG	ACAGAGTCGTCATCCGTCAC
Nrg4	CCCAGCCCATTCTGTAGGTG	ACCACGAAAGCTGCCGACAG
Adipoq	GTTCTACTGCAACATTCCGG	TACACCTGGAGCCAGACTTG
Adrb3	TGCGCACCTTAGGTCTCATTATGG	AAACTCCGCTGGGAACTAGAGAGG
Pgc1a	CCAGCCTCTTTGCCCAGATC	CGCTACACCACTTCAATCCACC
Cidea	TGACATTCATGGGATTGCAGAC	GGCCAGTTGTGATGACTAAGAC
Ucp1	GTGAACCCGACAACTTCCGA	TCCAGCGGGAAGGTGATG
F4/80	TCCAGAAGGCTCCCAAGGATA	GGGCACTTTTGTTCTCACAGGTA
Clec10a	GAGCTAGGAGCTCTCGCCAAG	GTCCCCAGTAATGTTGAGACCG
Il10	GCTCTTACTGACTGGCATGAG	CGCAGCTCTAGGAGCATGTG
Ym1	CCC CTGCCTGTGTACTCACCT	TCAATGCTTCATAGTCACGCAAGT
Arg1	CCCCAATGGTCAGGTTGATTC	CGTTTTCCATTAGCTCCTTCATGAT
Nos	GTTCTCAGCCCAACAATACAAGA	GTGGACGGGTCGATGTCAC
Il1b	GCAACTGTTCCTGAACTCAACT	ATCTTTTGGGGTCCGTCAACT
36b4	GCTCCAAGCAGATGCAGCA	CCGGATGTGAGGCAGCAG
